# Study protocol of sustaining home palliative care for patients with Heart Failure (HF) and their family caregivers in rural Appalachia: a mixed methods randomized clinical trial

**DOI:** 10.1186/s12904-025-01680-y

**Published:** 2025-03-07

**Authors:** Ubolrat Piamjariyakul, Stephanie Young, Angel Smothers, Sijin Wen, R. Osvaldo Navia, George Sokos, Ann E. Hendrickson, Peggy Fink, Diana Niland, Matthew Hottle, Angelo C. Giolzetti, Carol E. Smith

**Affiliations:** 1 West Virginia University, School of Nursing, Office 6701, Post Office Box 9600, Morgantown, WV USA; 2 Department of Biostatistics School of Public Health, West Virginia University, 64 Medical Center Drive, P.O. Box 9190, Morgantown, WV USA; 3 Division Chief of Geriatrics, Department of Medicine, West Virginia University, Palliative Medicine and Hospice, 64 Medical Center Drive, P.O. Box 9100, Morgantown, WV USA; 4 Interim Chair Department of Cardiology, West Virginia University, School of Medicine, and WVU Heart & Vascular Institute and Service Line, WVU Medicine, 1 Medical Center Drive, Morgantown, WV 26506 USA; 5 West Virginia University, School of Nursing Beckley Campus, 410 Neville Street, 101G Life Sciences Building, Room 101G, Beckley, WV 25801 USA; 6 West Virginia University, School of Nursing Keyser Campus, 101 Fort Avenue, Keyser, WV 26726 USA; 7 Department of Behavioral Medicine and Psychiatry, School of Medicine, Department of Behavioral Medicine and Psychiatry, West Virginia University, School of Medicine, Charleston, USA; 8 School of Nursing and School of Preventive Medicine, University of Kansas Medical Center, 3901 Rainbow Boulevard, Mail Stop 2029, Kansas City, KS 66160 USA

**Keywords:** Heart failure, End-of-life, Palliative care, Study protocol, Randomized controlled trial, Appalachia

## Abstract

**Background:**

Heart failure (HF) is the leading cause of mortality, morbidity, and rehospitalization in Appalachia. Rural areas have the highest HF mortality rates. Rural Appalachians lack access to health services and end-of-life palliative care (EOLPC) and have extreme inequities in health.

**Methods:**

The aim of this mixed methods randomized controlled trial (RCT) is to test the integrated nurse-led intervention bundle of the HF home EOLPC (**HF**-FamPAL**home**CARE) and to assess its ability to maintain sustainability with rural stakeholders, visiting volunteers, and the WV Faith Community Nurse Network. The participants are adult patients (50 to 80 years) with HF (NYHA III and IV and Stages C and D) and their caregivers (≥ 45 to 80 years).

The primary aim is to test the outcomes of patients with HF and family caregivers (104 dyads) managing home supportive EOLPC in rural WV. The secondary aim is to assess the bundled intervention for helpfulness, cost and sustainability. All participants received standard care from their regular providers. The intervention group received 2 home visits, 3 biweekly telephone calls and telephone reinforcement across 12 months. Data collection for both groups was conducted at baseline and at 3, 6, 9, and 12 months.

**Discussion:**

This RCT supports research to improve health equity by improving access to health services and addressing social determinants of health in underrepresented rural Appalachia. It is designed to test practical, sustainable approaches using available local resources to address HF symptom management, support EOLPC preferences, support older adults’ functional health and HF home caregiving skills, and provide social support.

**Trial Registration:**

ClinicalTrials.gov NCT06791850 Registered on date 19 January 2025.

## Background

Heart failure (HF) affects nearly 8 million Americans [1] and is increasing at a rate of 46%, with escalating costs of up to $70 billion annually [2]. HF is the leading cause of mortality, morbidity, and rehospitalization in Appalachia [3]. Rural areas have the highest HF mortality rates [4]. West Virginia (WV), the only state totally within the Appalachian region, has the highest HF death rate in the U.S. (32.6 per 100,000), with the greatest number of deaths occurring in those over age 65 [5]. HF is devastating for patients and their family caregivers, especially during the end stages [6]. Families are unprepared for this deteriorating condition, the burden of home caregiving, and the fear of painful death [7]. In addition, rural Appalachians lack access to health services and end-of-life palliative care (EOLPC) [8] and experience extreme health inequities [9]. Furthermore, physical and social isolation [10] is common due to the lack of/difficulty with transportation in WV’s rural mountainous environment, with limited internet or cellular services [9].

The goals of palliative care are to prevent and alleviate suffering and to enhance the quality of life for both patients and their families through a team-based, patient-focused approach to managing the discomfort, symptoms, and stresses associated with serious illnesses. This is achieved through accurate assessment, identification, and referral and through the treatment of pain and other symptoms, whether they are physical, psychological, or spiritual [11].

This RCT addresses the National Institutes of Nursing Research (NINR) strategic initiatives to identify ways to improve health outcomes and health equity in rural areas [12]. It also addresses the goals of the National Palliative Care Research Cooperative (PCRC) [13] and the EOLPC priorities of national organizations in advocating for the nursing community and for patients, families, and caregivers across all aspects of heart failure care [14].

Our preliminary RCT enrolled patients with HF and their family caregivers (N = 78) in the family palliative care (FamPALcare) preliminary trial. Compared with controls, both patients and their family caregivers had significant improvements in health measures and EOLPC outcomes at 6 months [15]. Additionally, the PI’s recent project, ‘Appalachian Visiting Neighbors,’ was tested with 30 older adults living alone. The results revealed that these older adults were able to improve their functional health by the 3-month follow-up. Both interventions were empirically verified as feasible and sustainable [16]. For both studies, Appalachian faith community nurses and trained visiting volunteers implemented all intervention visits and reinforced follow-up telephone calls. In practice, these nurses coordinated access and free travel to regional health care.

Thus, this RCT aims to test the integrated nurse-led intervention bundle of 1) HF home palliative care (**HF**-FamPAL**home**CARE), 2) visiting volunteers, and 3) Appalachian faith community nurses. This intervention bundle aims to address rural disparities in access to health care with the help of faith community nurses and local visiting volunteers [17, 18]. The intervention bundle components began with a series of preliminary studies while collaborating with Appalachian rural, religious, and policy leaders on our Rural Stakeholders Advisory Board. Our intervention’s bundle components are designed to mitigate Appalachian health disparities [19] by addressing critical social determinants of health (SDoH), such as faith community nurses reducing costs by enabling direct access to available health services, utilizing clearly illustrated HF home care guides for low literacy [20], and inspecting for safe housing for older adults [21].

## Conceptual framework guiding the HF-FamPALhomeCARE in this RCT

This RCT is guided by the social support conceptual theory confirmed by conceptual reviews and meta-analyses [22, 23]. Social support, such as helping neighbors, is a tenet of rural Appalachian culture [24]. This framework consists of four social support constructs, including emotional, informational, instrumental, and affiliation support [25]. Emotional support is described as providing caring listening, which can help decrease depression/anxiety [26]. Informational support is the process of knowledge transfer, which includes providing health information and instruction on specific HF home caregiving skills [25]. Instrumental support improves the in-home family caregiving burden by providing access to health care through the WV Faith Community Nurse Network and by organizing assistance [27] with daily needs such as shopping or transportation [28] utilizing local community resources. Affiliation support includes engaging in functional well-being and social activities with visiting volunteers to decrease social isolation [29]. Thus, social support and the intervention components are directly linked to patient and caregiver outcomes.

## Methods/design

### Aims

The aims of this RCT are to test the outcomes of the **HF**-FamPAL**home**CARE bundled intervention, with patients and family caregivers (104 dyads) managing home supportive EOLPC for end-stage HF in rural WV. The specific aims and hypotheses, with outcome measures for this RCT, are listed in Table 1.

**Table 1:** Specific Aims, Hypotheses, with the Outcome Measures for this RCT

**Primary Aims** are to test the outcomes of the **HF**-FamPAL**home**CARE bundled intervention, with patients and family caregivers managing home supportive EOLPC for end-stage HF in rural WV
**Aim 1:** Compared to the control group at 3, 6, 9 months, and 1-year follow-up:
***Hypothesis 1a.*** The patients in the intervention group will have higher scores on HF-related health quality-of-life measure and on the functional health well-being scale
***Hypothesis 1b.*** The family caregivers in the intervention group will have higher scores on the functional health well-being scale
**Aim 2:** Compared to the control group at 3, 6, 9 months, and 1-year follow-up:
***Hypothesis 2a.*** The patients in the intervention group will have (a) lower depression and anxiety scores and (b) higher numbers of signed EOL advance directives
***Hypothesis 2b.*** The family caregivers in the intervention group will have (a) lower depression and anxiety scores, (b) lower home caregiving burden scores, and (c) improved home HF EOLPC preparedness and improved home palliative care scores
**Secondary Aim** (Aim 3) is to assess the bundled intervention helpfulness, cost, and the plans for maintaining the sustainability of our faith community nurses and visiting volunteers engagement across rural WV
***Aim 3a.*** Compare the bundled intervention helpfulness questionnaire ratings by the patient, family caregiver intervention group participants, and the interventionists (faith community nurse and visiting volunteers). Determine the implementation cost of the **HF**-FamPAL**home**CARE bundle
***Aim 3b.*** Using focus group research, determine 10-year plans for sustaining the bundle implementation, maintaining rural volunteers and coordinating administrative aspects of the program

### Design/methodology

**Fig. 1:**
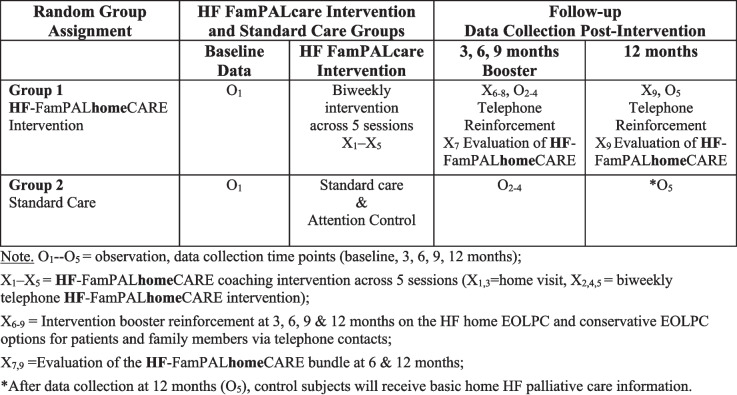
HF-FamPALhomeCARE Coaching Intervention (X_1_ to X_9_) Sequence with Data Collection (O_1_–O_5_)

This study uses a randomized controlled trial (RCT) design stratified by sex to determine the effectiveness and helpfulness of the **HF**-FamPAL**home**CARE bundled intervention (N = 208, 104 dyads: 104 patients and 104 family caregivers). The intervention group (52 dyads) will receive the intervention. The control group (52 dyads) will receive attention control consisting of education on general health topics that indirectly affect HF (e.g., vaccinations, handwashing). At the end of the 12-month completion period, each group received the content provided to the other group. See Fig. 1.

#### Randomization

The consulting statistician will use computerized randomization to generate numbers for subject enrollment. Two separate randomization lists will be created: one for males and one for females. Families will then be randomly assigned to either the control or intervention group at a 1:1 ratio. The group assignments are placed in sealed envelopes according to sex. Our RCT design adheres to CONSORT standards and rigorous research procedures [30]. The study is designed to enhance both internal and external validity, making it suitable for detecting differences between groups in behavioral intervention trials [31].

#### Characteristics of the participants

Adult patients (50 to 80 years of age) with HF (NYHA III and IV and Stages C and D) and their caregivers (≥ 45 to 80 years of age) who are involved in daily home care will be recruited. Recent national statistics revealed that 34% of adults aged 50 years or above had Stage B HF, which progresses to end-stages C and D within 5 years [32]. The inclusion age range for patients (50 to 80 years of age) is scientifically justified based on the 2023 National Heart Failure statistics from the American Heart Association (Chapter 22) [1] and our recent population-based heart failure study using 37,872 HF patients seen at WVU hospitals between 2008 and 2020 [33]. The rationale for the caregiver age range is based on our recent caregiving RCT study in Rural Appalachia [15] and two systematic reviews [6, 34] that included a total of 74 studies. These studies reported that the majority of caregivers’ ages ranged from 45 to 80 years.

#### Eligibility criteria

Patients will meet the inclusion criteria if they have advanced systolic and/or diastolic HF and have an informal caregiver aged 45 to 80. They do not need to live in the same home. Cardiology consultants specify the inclusion of patients with both systolic and diastolic dysfunction, as both require similar HF home care management regimens and have advanced EOL symptoms and palliative care needs [14]. Caregivers are those designated by the HF patient as a nonpaid primary person who assists with HF home care, thus not requiring caregivers to be spouses. All participants must be cognitively intact, able to provide written consent, and able to read and write in English. Each patient and his or her caregiver will be randomly assigned to a study group as a dyad.

Patients will be excluded if they have received or are on a waiting list for a heart transplant, have a left ventricular assist device, or have another terminal illness or dementia. Caregivers will be excluded if they have a disability that precludes their use of **HF**-FamPAL**home**Care intervention materials such as dementia [35].

#### Sample size

The power and sample size determination is based on a preliminary RCT, which revealed a moderate effect reduction in the burden of caregiving and improvements in patients’ HF-related health status [15]. With the use of G*Power v.3.1.9.7 for repeated-measures ANOVA with α = 0.05, Cohen’s d = 0.25 (moderate effect size), a power of 80%, two groups with 5 measurements, and correlations among repeated measures (0.50), a *sample size of 86 family dyads* will be needed. Our power analyses performed by this study’s statistician indicate that this sample size is sensitive enough to detect a significant difference in the study outcomes with this repeated measure design [36].

Our biostatistician’s revised precision calculation for linear mixed model analysis was based on the effect size outcomes of our HF palliative care studies. To account for an expected 20% attrition (many due to HF-related deaths), eight additional families will be recruited for each group. The calculated sample size was 86 patients, with an additional n = 18 for the expected 20% attrition rate (total sample N = 208; 104 patients and 104 caregivers) for this study. In this analysis, the random and fixed effects and quarterly repeated measures also add power.

#### Settings, recruitment and retention plans

The study will be conducted through the West Virginia University (WVU) School of Nursing. Recruitment will take place through WVU Medicine, various rural counties, health providers or clinics, provider offices, public meeting centers, and religious groups throughout Appalachia. We will utilize effective strategies for identifying and recruiting research participants in rural Appalachia [35]. Our designated nurse for participant enrollment will use IRB-approved invitations and established enrollment protocols to prospectively identify potential participants. Additionally, personnel with IRB-approved access to patient records will conduct subject eligibility screening and initial recruitment contact in compliance with HIPAA requirements. Our recruitment materials, including notices, brochures, flyers, postcards, and presentations, will use IRB-approved wording. Additionally, patients referred for HF palliative care consultations will be invited to participate if they meet the study criteria.

Given the large clinical population, rural stakeholder involvement, and our past study enrollment, we are confident in our ability to recruit patients and family caregivers for this study. The trained nurse recruiters will remain blinded to group assignments until after the random allocation of each patient‒caregiver dyad and the signing of consent [37]. There may be delays in enrollment during winter (December to March) caused by road conditions and extreme weather in rural Appalachia. The PI and Co-I will oversee participant timelines, monitor, and report enrollment progress on a quarterly basis.

Research studies conducted on the long-term maintenance of palliative care programs have revealed key characteristics for success and sustainability. These successes include involving well-respected locals (e.g., rural stakeholders, faith community nurses) [38] and designing scientific-specific care programs for a significant health concern. Additionally, addressing family members’ needs and incorporating the cultural traditions of the population served helps maintain programs [24]. Sustainability is supported by the use of resources compatible with health equity in marginalized rural areas [39]. Other factors for sustaining programs are the use of means that are well suited to the area for sharing ongoing information (i.e., radio, newspapers, religious bulletins, veterans, and farmer meetings).

Notably, each of these characteristics, known to lead to sustainability in care over time, are, in fact, integrated into our intervention. The scientific premise is that the ability to sustain comes from components within the intervention itself [40]. This premise also serves as our guide for addressing some of the SDoH by integrating these within the intervention components. For example, using local nurses and volunteers reduces costs and enables patients’ direct access to regional HF care. We are also using visual inspection for older adults’ safe housing, using graphics/photos illustrating HF home care guides for low literacy, and sharing available rural services [20]. Furthermore, as in the framework guiding this study, the intervention bundle is grounded in social support, which aligns with the Appalachian concept of caring for neighbors [24].

### Interventions

#### Standard care group

Participants in both groups will receive standard care, which is routine HF instruction at primary providers or specialist clinic appointments. Current standard HF instruction often has neither home HF skill-building, few home care instructions designed for family caregivers, nor EOLPC discussions. Standard care includes materials routinely given to all patients to achieve the national Joint Commission and AHA core HF standards for symptom management, support transitional care, and prevent 30-day readmission [41]. For time and attention control, the control subjects received instructions concerning handwashing techniques, vaccinations (e.g. Flu), and fraud prevention.

#### The HF-FamPALhomeCARE intervention bundle group

This study uses implementation science to deliver the verified evidence-based intervention bundle to the underserved population in rural Appalachia [39]. The **HF**-FamPAL**home**CARE intervention is a bundle of empirically verified preliminary HF trials [15] aimed at improving home HF palliative care via Appalachian-centered cultural approaches, engaging faith community nurses, and visiting volunteers to diminish health disparities and address the SDoH in rural Appalachia. The nurse interventionists in this RCT are experienced in arranging free travel to available rural/regional health care and WVU specialty care and are trusted by the medically underserved Appalachian population [42]. The nurse interventionist will select and train local volunteers to assist with home visits and telephone contacts. The intervention group will receive 5 biweekly sessions, which include two home visits (delivered by a team consisting of a faith community nurse and a visiting volunteer) and three biweekly telephone calls on HF home care, home safety, caregiver burden, and practical skills related to home EOLPC care.

We will use coaching and teach-back conversations during each intervention session to verify the understanding of HF education materials and practice home care skills [35]. Affirmations, positive statements, are used at the end of each session to encourage patients and their family caregivers to promote self-belief and motivation in HF home care [43]. For example, *“I will practice my skills to recognize HF symptoms, ways to improve fatigue, anxiety, and depression, and will report symptoms to the healthcare provider.”* Additional booster reinforcement sessions via telephone calls will be provided at 3, 6, 9, and 12 months to allow participants to practice home HF care skills and ask questions.

#### Treatment fidelity and quality assurance procedures

A comprehensive intervention manual with guiding scripts has been created. It includes strategies from previous family HF studies to facilitate **HF**-FamPAL**home**CARE discussions with each dyad. This manual will also help nurses build rapport by including content to address the anticipated needs and concerns of patients and family caregivers. Based on past trials and training roleplay, coaching sessions typically last between 60 and 120 min. Nurses are trained on using the **HF**-FamPAL**home**CARE manual and are advised to spend 30 min preparing for each session by reviewing session content [35]. Fidelity observations will be conducted and reported, with a positive fidelity rating achieved through consistent delivery of the intervention [44].

### Data collection procedure

#### Data collection for Aims 1 & 2

Data will be collected separately from all patients and caregivers at baseline and at 3, 6, 9, and 12 months. We will collect sociodemographic variables such as sex, age, race/ethnicity, comorbidities, and other social determinants of health (e.g., income adequacy, health insurance, education, health care services used, safe housing for older adults, etc.) at baseline. To decrease the risk of diffusion of treatment to the controls, the nurse interventionists working with families in the intervention group will be different from the nurses working with the control group. Those collecting data will be blinded to the group. Secure data collection and storage will be maintained. A HF home care checklist is used to assess the home care skills learned by the caregiver at 3, 6, 9, and 12 months and to identify areas that may need reinforcement. The interventionists and the participants in the intervention group completed separate anonymous helpfulness rating scales at 6 and 12 months to determine the helpfulness of the intervention bundle.

#### Data management and quality assurance

Data management protocols and an audit trail of the data management decisions will be kept for quality assurance and data integrity. Guides for verifying data and analyzing data distributional features and group equivalencies at the baseline will be followed [45]. Preliminary analysis will be conducted to verify that the statistical assumptions are met and whether transformations are needed. Differences in demographics between participants and those who declined to participate were reported. The reasons for declining to participate will be recorded.

To enhance the transparency and reproducibility of the research, the deidentified dataset, project data descriptions, and statistical models generated by R01 are shared [46]. These data will be shared in the appropriate NIH repository of the National Archive of Computerized Data on Aging (NACDA) hosted by the Inter-university Consortium for Political and Social Research (ICPSR). A description of the data management plan will also be submitted.

#### Managing missing data

Our biostatistician will review and discuss strategies for handling missing data, including assessing whether data are missing at random or repeatedly missing, and these strategies will be reported in publications [45]. Any missing data will be identified early and collected within two weeks. Research staff will use secure REDCap or Qualtrics surveys for data entry, with parameters set to identify ranges to monitor for missing data. Data will be converted from REDCap/Qualtrics to SPSS/SAS on a weekly basis. Data will be tracked in real time for subject enrollment, withdrawal, and dropout. The CONSORT enrollment diagram will be updated biannually and reported in research team meetings. The consulting biostatistician will guide the use of intent-to-treat statistical approaches and appropriate imputation methods (e.g., mean replacement) for all patient and caregiver outcomes.

#### Data collection for Aims 3a and 3b

*For Aim 3a,* each patient and family member independently rated the helpfulness of each intervention component via a 15-item Likert-type scale at 6 and 12 months after the completion of the **HF**-FamPAL**home**CARE intervention. The participants will rate their agreement with statements, with response options ranging from 1 (strongly disagree) to 5 (strongly agree). Sample statements include *“The nurse and volunteer prepared me for home HF care,” “I was comfortable during the discussion with the nurse/visitor,”* and *“I know ways to reduce breathlessness.”* Descriptive analysis will be used to evaluate the responses on the helpfulness scale. The nurse interventionists and visiting volunteers will rate the same helpfulness scale at 6 and 12 months following the **HF**-FamPAL**home**CARE intervention.

*Aim 3b: Focus group data collection.* The Principal Investigator (PI) and Co-Investigator (Co-I) are experienced in focus group research methods and working with families managing HF [47]. We will conduct 4 to 6 focus groups across West Virginia counties, each consisting of 3 to 5 rural stakeholders, such as church leaders, patients, and caregivers. These sessions will be held at rural sites, with the option of using a secured ZOOM for recording encrypted discussions and enabling participation from other rural leaders.

The PI and Co-I will facilitate discussions, starting with questions such as *“How can we collaborate to develop a 10-year plan for maintaining rural volunteers and continuing home care implementation?”* and *“What are the facilitators of and barriers to sustaining the HF-FamPALhomeCARE intervention in rural Appalachia?”* The same broad questions with prompts will guide all focus group discussions. The PI and Co-I will observe group dynamics to ensure that everyone participates and will ask probing questions for clarification and deeper insights.

The discussions will be audiotaped and transcribed, ensuring that no individual participants’ identities are disclosed. Each session will last approximately 60 to 90 min. Data saturation is achieved when no new topics emerge, with experts indicating that 99% of all new concepts typically emerge by the 20th participant [48]. All group data will be transcribed together.

### Data analyses

All the outcome measures have established discriminant or construct validity, internal consistency, reliability, and specificity. Each has been used with diverse populations, chronic illness patients, and HF families. These measures have been shown to distinguish clinically significant differences and are responsive to changes over time. These questionnaires were easily completed within 30 min. The sample demographics will be tabulated and reported.

#### Data analysis for Aims 1 & 2

Our experienced statistician will employ hierarchical generalized linear models with repeated measures to identify significant differences in outcomes between groups and to control for nested dyadic analysis [49]. The models will include fixed effects of treatment, time, and the treatment‒time interaction. Patient and family caregiver effects will be treated as random to account for dependence among repeated observations on the same subject as well as among subjects within the same family [37]. These models detect changes in outcome variables over time, differences between groups, and within-group differences over time. The SAS Proc MIXED procedure will be utilized. General linear modeling using linear contrasts of selected model parameters will test for significant differences. Point estimates and confidence intervals will be produced via contrasts and estimates of appropriate linear combinations of model parameters. The overall experimental error rate will be controlled via the Bonferroni procedure for multiple testing.

Multicollinearity among study variables will be determined and statistically controlled for in the data analysis. The number of mental health visits (for severe anxiety and depression) will be tracked for comparison between groups. To assess differences in HF EOLPC decisions, the number of family members and patients who sign an advance directive form, reveal a decision, or identify someone to help with HF EOLPC options will be recorded and reported. The proportion of patients signing an advance directive form will be used to evaluate the acceptability of EOLPC discussions. Subjects’ outcomes will be recorded at the time of death or 12 months postintervention, whichever comes first.

#### Data Analysis for Aim 3

##### Data analysis for Aim 3a

A descriptive analysis will be used to summarize the responses to the helpfulness scale items. Statistical assumptions will be tested to determine whether to use analysis of variance (ANOVA) or the Kruskal‒Wallis test for comparing mean scores between four groups: patients, caregivers, and interventionists (faith community nurses and visiting volunteers). Interventionists will also use anecdotal notes to report topics brought up by families that require reinforcement. These topics will be tabulated and reported to improve the intervention.

To determine the cost of the **HF**-FamPAL**home**CARE intervention implementation, all charges related to implementation are calculated. The items include the costs of personnel time for training and administering each discussion session, the materials, home/clinic visit travel, and telephone and mailing costs. Research costs related to data collection and material photocopying will be totaled and reported as study costs. The average intervention implementation cost across all families will be calculated and reported [15].

##### Data analysis for Aim 3b

We will conduct focus group research with rural stakeholders and our interventionists to create a 10-year plan for sustaining rural volunteers, continuing bundle implementation, and managing the administrative aspects of the program. Each focus group begins with an expression of gratitude for discussing the sustainability of the intervention bundle.

Content analysis will be used to transcribe discussions into written narrative data, with qualitative data analysis conducted continuously alongside data collection [50]. The PI and Co-I code the data after all focus group sessions, maintaining an audit trail of key decisions. Transcribed notes will be anonymized, and qualitative software will facilitate data management. Themes are compared with the social support framework, quantitative results, and Aim 3a helpfulness ratings to identify additional facilitators or barriers to sustainability [51].

To maintain the rigor of qualitative data analysis, the PI and Co-I independently code transcribed data and then reconcile categories. Themes are labeled via participants' words, and consistent questions across groups enhance credibility. New themes will provide insights into data dependability. Investigators will ensure 100% agreement on codes to avoid bias. Themes represent sustainability planning factors and are compared with quantitative measures, helpfulness results, and anecdotal notes from faith community nurses and visiting volunteers. Member checks involve rural stakeholders reviewing sustainability reports to ensure accuracy. These methods aim to achieve multiperspective 10-year planning solutions.

## Human subject protection and management of risks to subjects

Subject enrollment will occur across West Virginia and neighboring rural counties. Research staff will be trained and certified in Human Subjects Protection. The participants will provide consent, and a HIPAA waiver will allow access to the recruitment list. Confidentiality will be maintained through WVU policies, with measures in place to prevent breaches. Data management procedures include securely storing questionnaires and consent forms, and electronic data are stored on a firewall-protected server with daily backups.

## Data and safety monitoring

All adverse events will be reported to the IRB and the National Institute of Nursing Research (NINR). The PI and Co-Is review adverse events within 2 business days. Adverse events must be reported to the IRB within 3 business days. The PI will report IRB actions to the NINR within 2 business days. Any changes or amendments to the protocol or consent form will be reported to the NINR within 3 business days of IRB approval. Any change in scope will be submitted to the NINR for approval before implementation. Adverse events will be documented and reported to the NINR through the annual reporting system.

The Safety Monitoring Committee (SMC) for this RCT includes two independent experts. The SMC reviews each potential adverse event (AE) in real time and biannually. They provide guidance on interim analyses and stopping guidelines and address ethical issues related to palliative care, study safety, and research risks. Recommendations and actions will be reported to the IRB and NINR.

## Limitations and alternatives

The population of West Virginia is 93% Caucasian, but we have successfully enrolled underrepresented minorities in past studies. We anticipate a 20% attrition rate, primarily due to HF-related deaths. In previous studies, few caregivers withdrew after patient death, indicating that they were able to contact study nurses. To increase enrollment, program brochures are distributed in local churches by lay ministers and faith community nurses. Additionally, healthcare providers involved in HF patient discharges or ER visits may refer families to the program. Finally, the West Virginia University (WVU) integrated data repository (IDR) will be used for enrollment, as it has proven helpful for screening patients with HF across WV counties in the PI’s previous trials.

## Discussion

The aim of this mixed methods randomized controlled trial (RCT) is to test the integrated nurse-led intervention bundle of **HF**-FamPAL**home**CARE and to assess the bundled intervention helpfulness as well as develop plans for maintaining sustainable relationships with our rural stakeholder network, visiting volunteers, and the Faith Community Nurse Network of rural WV.

Notably, patients with HF and their caregivers in rural settings lack sufficient guidance for managing HF symptoms. Appalachians have expressed their desire to have access to health care and to die at home [8]. Thus, the **HF**-FamPAL**home**CARE intervention has been designed to address these issues by using Appalachian faith community nurses to engage families in culturally sensitive skills to manage HF symptoms, anxiety and depression and to hold discussions of their family’s EOL care preferences [52]. The home EOLPC discussed with families is based on HF provider recommendations and evidence-based national clinical guidelines for end-stage HF [14]. EOLPC discussions have resulted in care aligned with patient preferences, positive family evaluations of healthcare, and significantly fewer unwarranted HF hospital readmissions [53].

This R01 study supports research to improve health equity by improving access to health services and addressing disparities related to SDoH in underrepresented rural Appalachia [4, 8, 9]. Designing and testing practical sustainable approaches using available rural resources will address a prevalent disease, support EOLPC preferences, reinforce older adults’ functional health and HF home caregiving skills, and provide social support. Engaging rural stakeholders in recruitment and implementation and designing sustainability plans can result in continuing research on health and EOLPC and targeting disparities related to SDoH among the 26.3 million people living across Appalachia [9]. The long-term impact will be the creation of pragmatic strategies for other rural Appalachian states [54].

The PI and research team will adhere to the NIH Policy on Dissemination of NIH-Funded Clinical Trial Information. Clinical trial details and study results will be promptly updated and made publicly accessible via ClinicalTrials.gov, a database managed by the National Library of Medicine (NLM) of the National Institutes of Health (NIH). The trial results will be submitted within one year of the trial's primary completion date.

## Conclusion

The scientific merit of this study is that it is based on the social support framework, practices, and beliefs of the underserved rural Appalachian population, and the empirically verified research results from our series of preliminary HF palliative care studies. Our interventions address the needs of patients and family members while incorporating cultural traditions. Key factors for successful implementation and sustainability include the involvement of local nurse interventionists, the engagement of rural stakeholders, and the use of local resources to address health disparities and strengthen communities. This project will provide insights into sustaining volunteer programs in underserved Appalachian areas.

## Data Availability

No datasets were generated or analysed during the current study.

## Abbreviations

HF: Heart failure
EOLPC: End-of-life palliative care
HF-FamPALhomeCARE: Family home palliative care intervention for heart failure
IRB: Institutional Review Board
WV: West Virginia
SMC: Safety Monitoring Committee
AHA: American Heart Association
CONSORT: Consolidated Standards of Reporting Trials
SPIRIT: Standard Protocol Items: Recommendations for Interventional Trials
